# An Electrochemical Method to Detect Gamma Glutamyl Transpeptidase

**DOI:** 10.3390/ijms13032801

**Published:** 2012-03-02

**Authors:** Guifang Chen, Shengfa Ni, Sha Zhu, Jinghua Yang, Yongmei Yin

**Affiliations:** 1 Laboratory of Biosensing Technology, School of Life Sciences, Shanghai University, Shanghai 200444, China; E-Mail: gfchen@shu.edu.cn; 2 Department of Oncology, the First Affiliated Hospital of Nanjing Medical University, Nanjing 210029, China; E-Mails: inzo@163.com (S.N.); nanyizhusha@126.com (S.Z.); 3 Department of Biochemistry and State Key Laboratory of Pharmaceutical Biotechnology, Nanjing University, Nanjing 210093, China; E-Mail: josephinenju@yahoo.com.cn

**Keywords:** gamma glutamyl transpeptidase, glutathione, copper ions, electrochemical

## Abstract

Gamma glutamyl transpeptidase (GGT) is a transferase, which is of great importance in sustaining intracellular cysteine and glutathione levels. The abnormal expression of GGT is significantly associated with features of many metabolic syndromes (e.g., hepatocellular carcinoma). Therefore, it is essential to develop methods to detect GGT so as to monitor the physiological or pathological phenomena related to this species. In this work, by making use of a complex formed by Cu^2+^ and glutathione, which may exhibit excellent voltammetric response, we have proposed a novel potential electrochemical method for the detection of the enzyme. Results show that in the presence of GGT, the formation of Cu^2+^-glutathione complex on a working electrode will be disrupted, resulting in greatly depressed electrochemical signals. The primary method exhibits some advantages, such as it being fast, cost-efficient, and conveniently operated. It also has the potential to be further developed as an effective method in the quantitative detection of GGT in real samples.

## 1. Introduction

Gamma glutamyl transpeptidase (GGT) is a cell membrane-bound enzyme, which is located on the outer surface of cell membranes. It catalyzes the transfer of the gamma-glutamyl moiety of glutathione (GSH) to an amino acid, a peptide or water [1]. GSH, a tripeptide consisting of glutamine, cysteine and glycine, is produced in almost all cells. It is widely distributed in various tissues and performs many important physiological functions [2,3]. GGT plays a key role in the metabolism of GSH, and significantly contributes to sustaining intracellular cysteine and glutathione levels [4]. It is also involved in cancer drug resistance when overexpressed [5,6], and the over expression may also provide cancer cells with survival advantages under stress conditions [7]. Moreover, GGT-mediated metabolism of extracellular GSH is an important source of cysteine for tumor cells to resynthesize GSH. The increased expression of GGT is generally interpreted as a factor that favors the growth of several tumors [8]. Therefore, elevation of GGT is significantly associated with the features of many metabolic syndromes [9], and the detection of the enzyme may have important clinical applications. For example, hepatocellular carcinoma (HCC), which is frequently the long term result of viral hepatitis B (HBV) or C (HCV) infections associated with chronic inflammation and cirrhosis, is one of the most common fatal malignancies in the world. About 90 percent of tumors originating from liver are HCC. Although the diagnosis and treatment methods of HCC have been developing over the last 20 years, the recurrence and mortality of HCC is still high. One reason is that patients with HCC in the early stage are asymptomatic, and most of them are perhaps in the late-stage, advanced, or micro metastasis state when they are diagnosed [10]. So, early diagnosis is as important as efficient treatment and the understanding of the pathogenic mechanism of HCC.

It is known that some serum inflammatory markers are strongly related to HCC prognosis [11–15]; GGT is one of them, and has been considered for the evaluation of chronic hepatitis activity [16]. Some studies from HBV or HCV-related HCC have suggested that high levels of GGT are related to high incidence of HCC development, recurrence and poor prognosis [17]. Patients with HCC or some other hepatobiliary diseases are usually diagnosed to have high levels of GGT in their serum [18,19]. The serum activity of GGT may be even used as a diagnostic marker of liver cancer [20]. Owing to the importance of GGT, some available methods have been developed to detect the enzyme. Spectrophotometric measurement [21,22], which is recommended by the International Federation for Clinical Chemistry and Laboratory Medicine (IFCC), is the most prevailing method, and has been adopted in clinics. Besides fluorescence [23], high-performance liquid chromatography (HPLC) [24], and electrophoresis [25] have also been successfully employed in the detection of the activity of GGT. Though the quantitative detection of GGT *in vitro* has thereby been achieved, some disadvantages still exist. For example, electrophoresis and HPLC methods are relatively time consuming and inconvenient, while spectrophotometric and fluorescent measurements need costly apparatus and some toxic chemical reagents (e.g., 5-amino-2-nitrobenzoate), which restrict the development of portable home medical devices. Electrochemical technique has been well known for its fast detection, easy operation, low cost, high sensitivity, and easy-to-miniaturize. In this work, we propose a novel primary electrochemical method to detect GGT, which may be further developed as an effective method in the quantitative detection of GGT in real samples.

## 2. Results and Discussion

**Figure 1:**
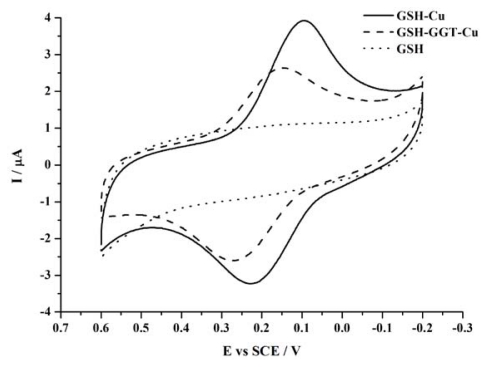
Cyclic voltammograms obtained at the glutathione (GSH) modified electrode before (dot line) and after (solid line) it is treated with 1 μM Cu^2+^ for 1 h. The dash line shows the case that the GSH modified electrode has been previously incubated with 5 U/mL GGT at 37 °C for 30 min before the treatment of Cu^2+^. Scan rate: 50 mV·s^−1^.

We first immobilized the substrate GSH onto a gold electrode through an Au-S bond between the surface of electrode and the thiol group of GSH. On the other hand, copper ions have been proven to exhibit electrochemical signals by its complexation with some species, such as GSH [26], polyaspartate [27] and cysteine [28]. This finding is also confirmed by our results. As is shown in Figure 1, after complexation with Cu^2+^, the GSH modified electrode may show voltammetric peaks, whereas no redox waves can be observed in the absence of Cu^2+^. The redox peaks are attributed to the reduction and oxidation of Cu^2+^/Cu^+^ couples. Further results show that after the GSH modified electrode is treated with GGT, the voltammetric peaks of the electrode decrease.

The result is as expected, because under the catalysis of GGT, the glutamic acid moiety of GSH is cut off, leaving the cysteinylglycine moiety alone attached on the electrode, which cannot then form a complex with Cu^2+^. Therefore, after the incubation of the GSH modified electrode with the enzyme, the amount of Cu^2+^-GSH complex decreases, which results in the decreased electrochemical peaks.

**Figure 2:**
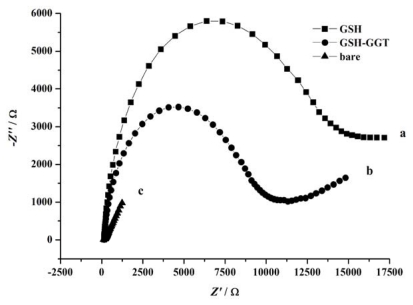
Electrochemical impedance spectra of GSH modified electrode: (**a**) before; (**b**) after its incubation with 5 U/mL GGT at 37 °C for 30 min. Curve (**c**) is in the case of bare gold electrode. 5 mM K_3_Fe(CN)_6_/ K_4_Fe(CN)_6_ working as electrochemical probe was added to the electrolyte, initial E was set at 0.224 V, amplitude was 0.005 V, high and low frequency were 10^5^ Hz and 0.01 Hz, respectively.

Electrochemical impedance spectrum (EIS) technique is employed to characterize the surface alteration of the gold electrode after its modification with GSH, and subsequently the catalysis by GGT. Because GSH is unfavorable for the electron transfer between the electrochemical probe [Fe(CN)_6_]^3−/4−^ and the electrode surface as a result of steric exclusion, after the modification of GSH onto the gold electrode surface, an increase of the electron-transfer resistance can be observed (Figure 2), representing a big semi-circle. However, after the catalysis by GGT, the glutamic acid moiety of GSH is cut off from the electrode and the steric exclusion decrease, representing a small semi-circle, whose diameter is between a GSH modified electrode and a bare electrode.

**Figure 3:**
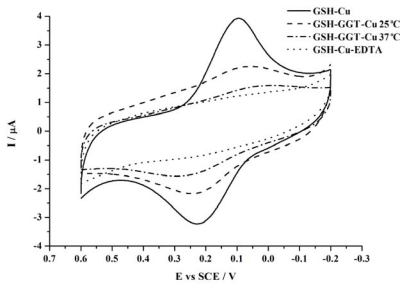
Cyclic voltammograms obtained at the GSH modified electrode in the presence of 1 μM Cu^2+^ for 1 h. The GSH has been pre-incubated with 5 U/mL GGT at 37 °C (dash dot line) or 25 °C (dash line) for 30 min, or without GGT (solid line), and then incubated with a gold electrode for 16 h to form the GSH modified electrode. The dot line shows the case that EDTA instead of GGT is added in the test solution. Scan rate: 50 mV·s^−1^.

We also conducted the experiments using an alternative strategy in which the enzyme catalyzed reaction takes place in solution rather than on the electrode surface. As has been described in the experimental sections, GGT is first allowed to catalyze the transfer of the glutamic acid moiety of GSH in a solution. The production containing glutamic acid and cysteinylglycine together with the enzyme itself is then incubated with a polished electrode, allowing the interaction between cysteinylglycine and the surface of the electrode. After further treatment with Cu^2+^, the electrode is measured by cyclic voltammetry (CV). As is shown in Figure 3, the peaks decrease more drastically than that in the former case (dash dot line in Figure 3 compared with dash line in Figure 1). The result is reasonable, since the solution environment provides more opportunities for the interaction between GGT and GSH than that on a surface, and most of GSH is cut off, consequently less Cu^2+^-GSH complex can be formed to provide electrochemical signals.

Besides, we conduct the experiments at 25 °C to further confirm that the current decrease is attributed to the GGT catalysis. As is also shown in Figure 3 (dash line), the current decrease is less prominent than that at 37 °C. Because the optimum temperature for GGT is 37 °C, the activity of GGT is limited at 25 °C, so as to show an expected experimental performance.

As is well known, Cu^2+^ is chelated with EDTA very strongly. So we make use of EDTA as an inhibitor for the formation of Cu^2+^-GSH complex. As is shown in Figure 3 (dot line), while EDTA instead of GGT is added, the current decrease is so apparent that no peaks can be observed anymore, suggesting that the chelation between Cu^2+^ and EDTA is more efficient than the catalysis of GGT.

**Scheme 1:**
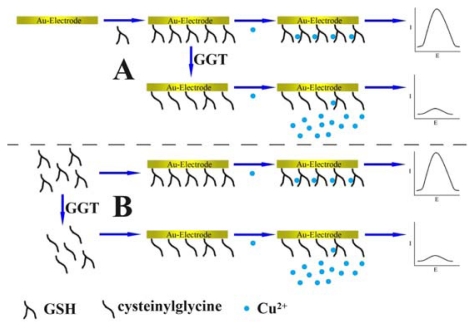
Schematic illustration of the electrochemical method to detect the activity of GGT. (**A**) A GSH modified electrode is first prepared. Then the GGT-mediated catalysis is allowed to occur on the surface of the electrode. Finally, Cu^2+^ is added in order to produce the electrochemical response; (**B**) GSH is first cleaved by GGT in a solution. Then GSH is linked to a gold electrode. Finally, Cu^2+^ is added in order to produce the electrochemical response.

The overall method for the detection of GGT is illustrated in Scheme 1. As is shown in this scheme, two strategies with slight differences are proposed. In Scheme 1A, GGT-mediated catalysis occurs on the surface of the electrode, while in Scheme 1B, the catalytic reaction takes place in the solution. Despite the difference, results show that both of the strategies work well. In the former case, the strategy is more convenient to operate, because only *ca*. 90 min is required to complete the detection at the introduction of the target GGT. The latter is otherwise better in signal behavior, since the GGT caused current changes are more drastic in this case. The choice of which one is better should depend on the needs and further tests. In comparison with the prevailing spectrophotometric method, the electrochemical method shown here has comparative operability, test time, and cost. A main disadvantage of the electrochemical method is that a modified electrode should be prepared, which requires hours (16 h in our experiments). Fortunately, it may be excluded in the test process (strategy shown in Schme 1A) and may be commercially mass-produced. On the other hand, the major advantage of the electrochemical method is that all the experimental materials are readily obtained and cost-effective without requiring the usage of toxic chemical reagents.

## 3. Experimental Section

### 3.1. Materials

GGT from equine kidney (9.1 mg, 100 Units) was purchased from Sigma and used without further purification. GSH was purchased from Nanjing Dazhi Biotechnology Co., Ltd. CuSO_4_, and EDTA were acquired from Nanjing Chemical Reagent Co., Ltd, which were both of analytical grade. CuSO_4_ was dissolved in 0.1 mM HNO_3_. All solutions were prepared with double-distilled water which was purified to a specific resistance (18.2 MΩ/cm) with a Milli-Q purification system (Barnstead, Asheville, NC, USA) and were stored in 4 °C for use.

### 3.2. Preparation of GSH Modified Electrode

Gold electrode, the gold diameter of which was three millimeters, was purchased from Ada Tianjin Science and Technology Development Co., Ltd. The gold electrode was firstly polished on fine sand papers and alumina (particle size of about 0.05 μm)/water slurry on silk. Then it was thoroughly ultrasonicated in ethanol and doubly distilled water for about 5 min, respectively. Finally, the electrode was electrochemically cleaned to remove any remaining impurities in 1 M H_2_SO_4_. After being dried with nitrogen, the gold electrode was immersed in 0.5 mL solution containing 3 mg/mL GSH for 16 h. The GSH could bind to the gold electrode through an Au-S bond between the surface of electrode and the thiol group of GSH. After thoroughly rinsed with pure water and dried with nitrogen, GSH modified electrode was prepared and could be ready for use.

### 3.3. GGT Catalyzed Reaction on the GSH Modified Electrode

The prepared GSH modified gold electrode was further immersed in a 0.5 mL solution containing 5 U/mL GGT at 37 °C for 30 min, during which the GGT would catalyze the transfer of the glutamic acid moiety of GSH, leaving the cysteinylglycine moiety alone attached on the electrode. After thoroughly rinsed with pure water, cysteinylglycine modified electrode was prepared. For comparison, the cysteinylglycine modified electrode could be also prepared by an alternative strategy, in which the enzyme catalyzed reaction took place in solution. In this strategy, a solution containing 5 U/mL GGT and 3 mg/mL GSH was first incubated at 37 °C for 30 min to allow the reaction occur. Then, a polished gold electrode was immersed in the solution for 16 h. After being thoroughly rinsed with pure water, the cysteinylglycine modified electrode was prepared.

### 3.4. Electrochemical Measurements

The GSH modified electrode or cysteinylglycine modified electrode was further immersed in a solution containing 1 μM CuSO_4_ for an hour before electrochemical measurements. Then, the electrode was thoroughly rinsed with pure water, and was ready for measurements. Electrochemical measurements by using CV and EIS techniques were performed on a model 660C Electrochemical Analyzer (CH Instruments) with a PBS buffer (100 mM, pH 7.0) working as electrolyte. The modified electrode was used as working electrode, and a saturated calomel electrode (SCE) and a platinum electrode were used as the reference and counter electrode, respectively. For CV test, scan rate was set at 50 mV/s, while for EIS, 5 mM K_3_Fe(CN)_6_/K_4_Fe(CN)_6_ working as electrochemical probe was added to the electrolyte, initial E was set at 0.224 V, amplitude was 0.005 V, high and low frequency were 10^5^ Hz and 0.01 Hz, respectively.

## 4. Conclusions

In summary, we have developed an electrochemical method to detect GGT based on the voltammetric response of the complex formed by Cu^2+^ and GSH. The detection can be easily and conveniently performed, and the preparation of the modified electrode is also simple without any troublesome procedures necessary. Therefore, the proposed method for the detection of GGT may have potential application in the diagnosis of liver cancer in the future.
